# Synchrotron XRF Analysis Identifies Cerium Accumulation Colocalized with Pharyngeal Deformities in CeO_2_ NP-Exposed *Caenorhabditis elegans*

**DOI:** 10.1021/acs.est.1c08509

**Published:** 2022-04-04

**Authors:** Lisa Magdalena Rossbach, Dag Anders Brede, Gert Nuyts, Simone Cagno, Ragni Maria Skjervold Olsson, Deborah Helen Oughton, Gerald Falkenberg, Koen Janssens, Ole Christian Lind

**Affiliations:** † Faculty of Environmental Sciences and Natural Resource Management, Norwegian University of Life Sciences, P.O. BOX 5003 NMBU, No-1432 Ås, Norway; ‡ Centre for Environmental Radioactivity (CERAD CoE), Faculty of Environmental Sciences and Natural Resource Management, Norwegian University of Life Sciences (NMBU), P.O. Box 5003, 1432 Ås, Norway; § Faculty of Science, AXIS Research group, University of Antwerp, Groenenborgerlaan 171, 2020 Antwerp, Belgium; ∥ Faculty of Natural Sciences, Norwegian University of Science and Technology, P.O. Box 8900, No-7491 Trondheim, Torgarden, Norway; ⊥ Photon Science, Deutsches Elektronen-Synchrotron DESY, Notkestr. 85, 22607 Hamburg, Germany

**Keywords:** *sod-1*, redox balance, elemental mapping, nanotoxicology, X-ray fluorescence

## Abstract

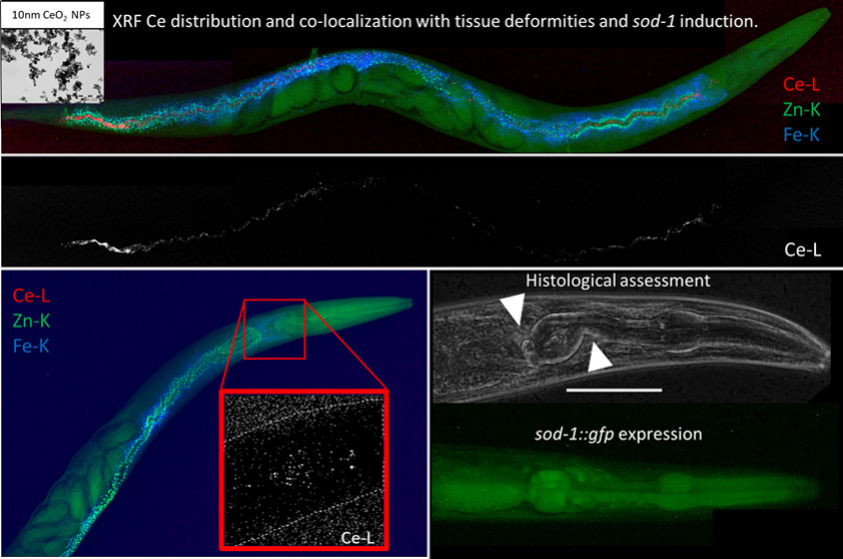

A combination of synchrotron radiation-based elemental imaging, in vivo redox status analysis, histology, and toxic responses was used to investigate the uptake, biodistribution, and adverse effects of Ce nanoparticles (CeO_2_ NP; 10 nm; 0.5–34.96 mg Ce L^–1^) or Ce(NO_3_)_3_ (2.3–26 mg Ce L^–1^) in *Caenorhabditis elegans*. Elemental mapping of the exposed nematodes revealed Ce uptake in the alimentary canal prior to depuration. Retention of CeO_2_ NPs was low compared to that of Ce(NO_3_)_3_ in depurated individuals. X-ray fluorescence (XRF) mapping showed that Ce translocation was confined to the pharyngeal valve and foregut. Ce(NO_3_)_3_ exposure significantly decreased growth, fertility, and reproduction, caused slightly reduced fecundity. XRF mapping and histological analysis revealed severe tissue deformities colocalized with retained Ce surrounding the pharyngeal valve. Both forms of Ce activated the sod-1 antioxidant defense, particularly in the pharynx, whereas no significant effects on the cellular redox balance were identified. The CeO_2_ NP-induced deformities did not appear to impair the pharyngeal function or feeding ability as growth effects were restricted to Ce(NO_3_)_3_ exposure. The results demonstrate the utility of integrated submicron-resolution SR-based XRF elemental mapping of tissue-specific distribution and adverse effect analysis to obtain robust toxicological evaluations of metal-containing contaminants.

## Abstract 2

The combination of SR-based μXRF tissue-specific elemental mapping and adverse effect analysis provides a robust toxicological evaluation of nanoceria.

## Introduction

Cerium dioxide nanoparticles (CeO_2_ NPs, or nanoceria) are among the most manufactured nanomaterials by mass due to their unique properties and application in vehicle catalytic emission control systems, electrolyte materials of solid oxide fuel cells, and ultraviolet-blocking materials.^1,2^ Hazard assessments show consistent results regarding the toxicity of cerium (Ce) ions for a range of organisms.^3−5^ Nevertheless, there are conflicting findings regarding the toxic effects of CeO_2_ NPs. Investigations of CeO_2_ NP toxicity indicate highly particle-specific modes of action (MoA), with the reported effects ranging from highly toxic to nontoxic and even beneficial effects.^6,7^ It has been proposed that the apparent differences originate from the intrinsic catalytic capacities that enable CeO_2_ NPs to act as a scavenger of superoxide and hydroxyl radicals, referred to as antioxidant enzyme-mimetic activity.^8−10^ Furthermore, the binding of hydrogen peroxide (H_2_O_2_) on the surface of the NPs in solution, with subsequent catalytic reduction to water (H_2_O) and oxygen (O_2_), has been shown to facilitate cellular antioxidant activity and hence avoid the production of ^·^OH by CeO_2_ NPs.^11,12^

Despite the reactive oxygen species-scavenging properties of nanoceria, a range of toxicological studies have shown adverse effects of the NPs in in vitro and in vivo models.^13−17^ In the nematode *Caenorhabditis elegans*, Roh et al.^15^ showed higher toxicity from smaller (15 nm) CeO_2_ NPs compared to that of larger (45 nm) particles. Moreover, CeO_2_ NPs (53 ± 3 nm) were more toxic than the equimolar bulk CeO_2_,^16^ while smaller (8 nm) CeO_2_ NPs lead to reactive oxygen species (ROS) accumulation and oxidative damage in metal and oxidative stress-sensitive transgenic nematode strains.^17^

*C. elegans* is, due to its transparency and relatively small size, a suitable model for in vivo redox sensor based on fluorescent probe measurements^18−22^ and imaging the distribution of ingested metals and metallic nanomaterials in the whole organism or individual organs.^16,23−26^ Whole body investigation by two-dimensional (2D) X-ray fluorescence (XRF) tomography at synchrotron beamline facilities, with submicron spatial resolutions, may therefore take advantage of these features to identify ingested NPs within tissues, organs, and eggs.^24^ Such noninvasive imaging techniques may provide vital information on internal distributions and interactions of metal NPs with tissues and organs and help explain toxic effects.^24^

Several studies have shown a nonhomogeneous distribution of various types of NPs in *C. elegans* including some reports of translocation of NPs into tissues surrounding the lumen.^24,27−29^ Moreover, Yang et al.^27^ related the biodistribution of citrate-coated Ag NPs (25 ± 9 nm), measured by hyperspectral imaging and TEM analysis, to swelling of intestinal cells and enlarged mitochondria. While the uptake of gold NPs by nematodes was confirmed by synchrotron radiation-based X-ray analysis in a study by Hu et al.,^28^ toxic effects, including changes in the locomotion, population survival, and gene expression, were not correlated with spatial distributions of Au.

Despite a range of studies reporting nanoceria-induced toxic effects, including increased ROS production, oxidative damages, and decreases in growth and fertility of nematodes, or beneficial effects, such as protective properties against oxidative-mediated apoptosis in cell cultures,^6,15−17^ there is a lack of studies relating observed effects to spatial distributions covering the whole organism. The current study aimed to investigate if exposure to CeO_2_ NPs or Ce(NO_3_)_3_ would lead to systemic uptake and whether tissue-specific Ce accumulations lead to adverse effects. To this end, we characterized uptake and whole organism biodistribution of CeO_2_ NPs and Ce(NO_3_)_3_ in *C. elegans* using submicron-resolution synchrotron radiation-based XRF elemental mapping. Furthermore, we examined the adverse effects, in terms of growth, fertility, and reproduction and oxidative stress development in *C. elegans* following the exposure to either CeO_2_ NP or Ce(NO_3_)_3_.

## Methods

### Nanoparticle Preparation and Characterization

The CeO_2_ NP (544841, Sigma-Aldrich) (10 nm, Figure S1) stock suspensions were prepared 24 h prior to the start of the exposures. Size distribution of the NPs was measured using transmission electron microscopy (TEM, Morgagni 268, FEI), nanoparticle tracing analysis (NTA, Nanosight LM10, Malvern Panalytical), and dynamic light scattering (DLS, Malvern PN3702 Zetasizer Nanoseries, Malvern Inc., Malvern, U.K.). Furthermore, size distribution measurements of the NPs in the exposure media were conducted at T 0 and 72 h. For further details, see Supporting Information section 1.1.

### Nematode Culture and Exposure

All *C. elegans* strains, N2 Bristol (Caenorhabditis Genetic Centre, Minneapolis), SOD-1 (GA508 wuls54[pPD95.77 sod::1GFP, rol-6(su1006)]) (Institute of Healthy Ageing Genetics, University College London), and HyPer and Grx1-roGFP2 (GRX),^19^ were exposed to either Ce(NO_3_)_3_ (2.3–26 mg Ce L^–1^) or CeO_2_ NPs (0.5–34.96 mg Ce L^–1^) in standard toxicity tests according to the International Organization of Standardization,^30^ with some modifications (see Supporting Information section 1.2).

Synchronized L1 stage nematodes were exposed for 96 h, and toxic effects on viability, growth, fertility, and reproduction were measured as described in Supporting Information section 1.2. For the in vivo assessment of cerium onto the redox homeostasis, the Sod-1, HyPer, and GRX strains, nematodes were sampled at 72 h of exposure. Samples were analyzed as previously described by Rossbach et al.^21^ and Maremonti et al.^22^

Cerium uptake by exposed nematodes was measured using inductively coupled mass spectroscopy (ICP-MS, ICP-MS Agilent 8800, Mississauga, Canada) measuring the Ce (140) isotope, at a detection limit of 0.000 3 ppm and limit of quantification of 0.000 9 ppm. Total body burden and retained cerium post depuration were measured from triplicate samples from three exposure concentrations of either Ce(NO_3_)_3_ (2.3, 4.19, or 6.14 mg Ce L^–1^) or CeO_2_ NPs (0.99, 8.03, or 34.96 mg Ce L^–1^) at 72 h (for further detail, see Supporting Information section 1.2).

### Nematode Preservation for SR Imaging Analysis

Cerium-exposed adult nematodes (4.17 or 17.48 mg L^–1^ of Ce(NO_3_)_3_ or CeO_2_ NPs, respectively) were gently transferred to a 15 mL Nunc tube, centrifuged (280*g*, 1 min) at room temperature, supernatant-aspirated, and washed once in 5 mL of moderately hard reconstituted water + Tween 20 (MHRW + T). Nematodes from selected samples were depurated by feeding on *Escherichia coli* OP50 NGM agar plates for 2 h and subsequently washed in 5 mL of MHRW—Telaranea nematodes were fixed using 2% PFA and 1% GA as previously described.^24^ The fixed nematodes were stored in PBS-buffer until SR analysis. Immediately prior to analysis, nematodes were mounted onto 0.25 cm^2^ frames with a 1 μm-thick silicon nitride membrane, covered by a 10 μm layer low-melting point agarose, and sealed by gluing a 4 μm-thick Ultralene film onto the frame.

### Micro-XRF Scanning

Microfocused synchrotron radiation-based XRF experiments were performed at the microprobe end station of the P06 Hard X-ray Micro/Nano-Probe beamline of the PETRA III storage ring of the DESY facility (Hamburg, Germany),^31^ using an excitation photon energy of 12 and 19.5 keV selected by means of a Si(111) double-crystal monochromator. A Kirkpatrick–Baez mirror optic was used to focus the beam to a spot size of about 0.8 × 0.8 μm^2^ (*h* × *v*). A Keyence optical microscope equipped with a perforated mirror allowed for positioning of the sample. Fluorescent X-rays were detected using the Maia detector array.^32^ Two-dimensional images were obtained by raster scanning the samples in 200–500 nm steps (horizontal and vertical) in the microfocused beam, while registering a full XRF spectrum for every pixel with 3–50 ms acquisition time. Stitching of the recorded maps was performed using inhouse developed software, Datamuncher.^33^ XRF spectral fitting was performed using the PyMCA software package.^34^

### Statistical Analysis

Statistical analysis of the data was performed using R 3.5.2 (R Core Team, 2018). Following descriptive statistical analysis, a one-way analysis of variance (ANOVA) was performed to check for statistically significant differences between groups. A post-hoc Tukey’s honestly significant difference (HSD) test was applied to find *p*-values between all group combinations. A significance level of 0.05 was used in all tests.

## Results and Discussion

### Whole Body Submicron Resolution of Elemental Distribution

The toxicity of NPs is highly dependent on the uptake and retention of particles by the organisms, where the internalized fraction has been shown to be more important as a toxicological indicator than the actual exposure concentration.^35−39^ Information on tissue interactions and potential adversity of the internalized NP fraction may thus provide critical information. In a previous study, we conducted nanoscopic elemental mapping to obtain whole body cobalt NP distribution, and NP tissue interactions in preserved and dehydrated *C. elegans*.^24^ In the current work, a sample preparation method designed to preserve the organism without potentially destructive dehydration steps was developed. This allowed for detailed whole body elemental mapping of intact nematodes under pristine conditions, with the detection of a range of essential elements including biometals such as zinc and iron and Ce and other potentially toxic metals and mapping their distribution at resolution down to 800 nm (Figure S2).

For the visualization of biological structures such as organs, tissues, and cells and abundant elements, such as Zn, Fe, or phosphorus, distribution maps were used as a reference. This allowed for the identification of the nematode’s intestine, pharyngeal structures, and reproductive organs (see Figure S3 A for annotation) and fine details such as the large somatic nerve ring surrounding the isthmus in the pharynx (Figure S3F).

### XRF Elemental Analysis Reveals Ingestion and Retention but No Translocation of Ce from CeO_2_ NPs from the Intestine into the Surrounding Tissues

**Figure 1:**
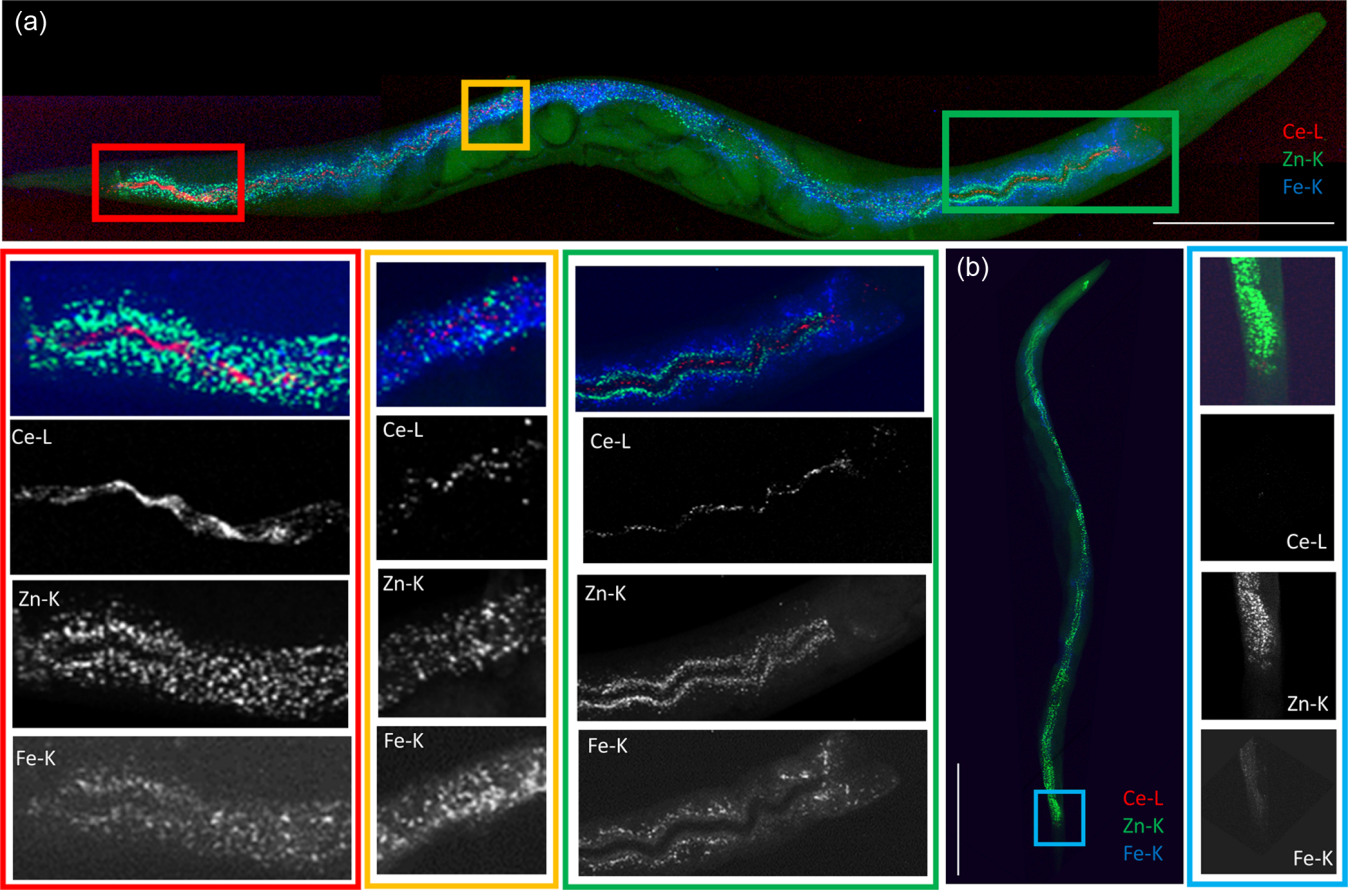
Two-dimensional XRF elemental distribution maps showing Ce accumulations in undepurated (a) (12 keV; 0.5 × 0.5 μm^2^ step size; 823 × 149 μm^2^ map size; 50 ms exposure/pt) and depurated (b) (12 keV; 0.2 × 0.2 μm^2^ step size; 569 × 802 μm^2^ map size; 3 ms exposure/pt) CeO_2_ NP exposed nematodes, including Ce (red), Zn (blue) and Fe (green) elemental map overlay. Colored boxes on whole nematode maps indicating the regions of interest with detailed Ce, Zn, and Fe elemental maps. Scale bars represent 100 μm.

While no Ce was detected in unexposed controls (Figure S3B), 2D XRF elemental mapping revealed Ce in the undepurated nematode exposed to 17.48 mg L^–1^ CeO_2_ NPs (Figure 1A). High levels of Ce were predominantly visible within the intestinal lumen, in particular the anterior and posterior part of the midgut. Elemental distribution patterns did not show any colocalization with either the Zn or Fe distribution maps (Figure 1B), indicating no translocation to Zn containing lysosomal granules. The lack of Ce signals outside the intestinal lumen suggested no further translocation of the NPs. However, the high abundance of Ce across the entire midgut could indicate a significant binding or interaction with the microvilli, intestinal epithelium, or the glycocalyx. Due to changes in the expression of various proteins, associated primarily with the intestine of *C. elegans*, a previous study has identified the gut lining as an important target for nanoceria toxicity.^40^ Similar to the current study, hyperspectral imaging of CeO_2_ NP in *C. elegans* indicated the presence of particles in the gut of the nematodes.^16^ Following the exposure to 12.5 mg Ce L^–1^ CeO_2_ NPs (53 ± 3 nm) in MHRW, high-density areas, believed to be particle aggregates within the gut of the nematode, were visible on hyperspectral dark-field images.^16^

Analysis of the Ce distribution revealed a few Ce signals associated with the exterior cuticle of the nematode (Figure 1). Therefore, to assess the extent of the retention and translocation, Ce distribution was mapped in a depurated nematode. The Ce exposure in the current study spanned across the entire developmental stages of the nematode (L1–L4), which is expected to facilitate translocation of NPs and hence should be observable within depurated nematodes. Nevertheless, the XRF maps revealed overall low Ce retention following depuration (Figure 1B). These findings suggest little direct interaction of the CeO_2_ NPs with epithelial membranes or microvilli. Despite the the negative charge (−10 mV) of the NPs in MHRW applied in the present study, which has been shown to facilitate cellular uptake of nanoceria,^41^ no evidence of Ce translocation across the intestinal barrier and into cellular compartments was detected. These findings are consistent with findings by Arnold et al.,^16^ who found a high degree of ingestion with little retention but no translocation of particles to luminal cells and different compartments. Notably, in the current study, the pharynx and the hindgut showed a low Ce content, with no detection in the midgut region of the depurated nematodes. This is unlike previous observations that showed high retention of Co NPs within and surrounding nematode intestines.^24^

The XRF mapping and ICP-MS measurements of undepurated nematodes were consistent. For depurated nematodes, however, the XRF mapping showed low retention, while ICP-MS analysis revealed greater variability related to different exposure concentrations (Table S1). Speciation results revealed little to no low-molecular mass (LMM) Ce fraction present in the exposure, suggesting a high stability of the particles. Therefore, particles may remain inert and have little potential for dissolution, interaction, or further translocation within the nematodes.

The low surface but high luminal Ce content reveals ingestion as the primary route of exposure for CeO_2_ NPs in nematodes. Pelletier et al.^42^ showed the interaction of the CeO_2_ NPs with the *E. coli* cell walls but no internalization by the bacteria. The high (65.4–79.5%) particulate (<0.45 μm) Ce fraction in the CeO_2_ NP exposure in the current study (Figure S4) may therefore be a result of interaction of the Ce with the *E. coli* cells, which could have facilitated ingestion of the Ce by the nematodes.

### Nonhomogeneous Distribution of Ce from Ce(NO_3_)_3_ in the Intestine of *C. elegans*

**Figure 2:**
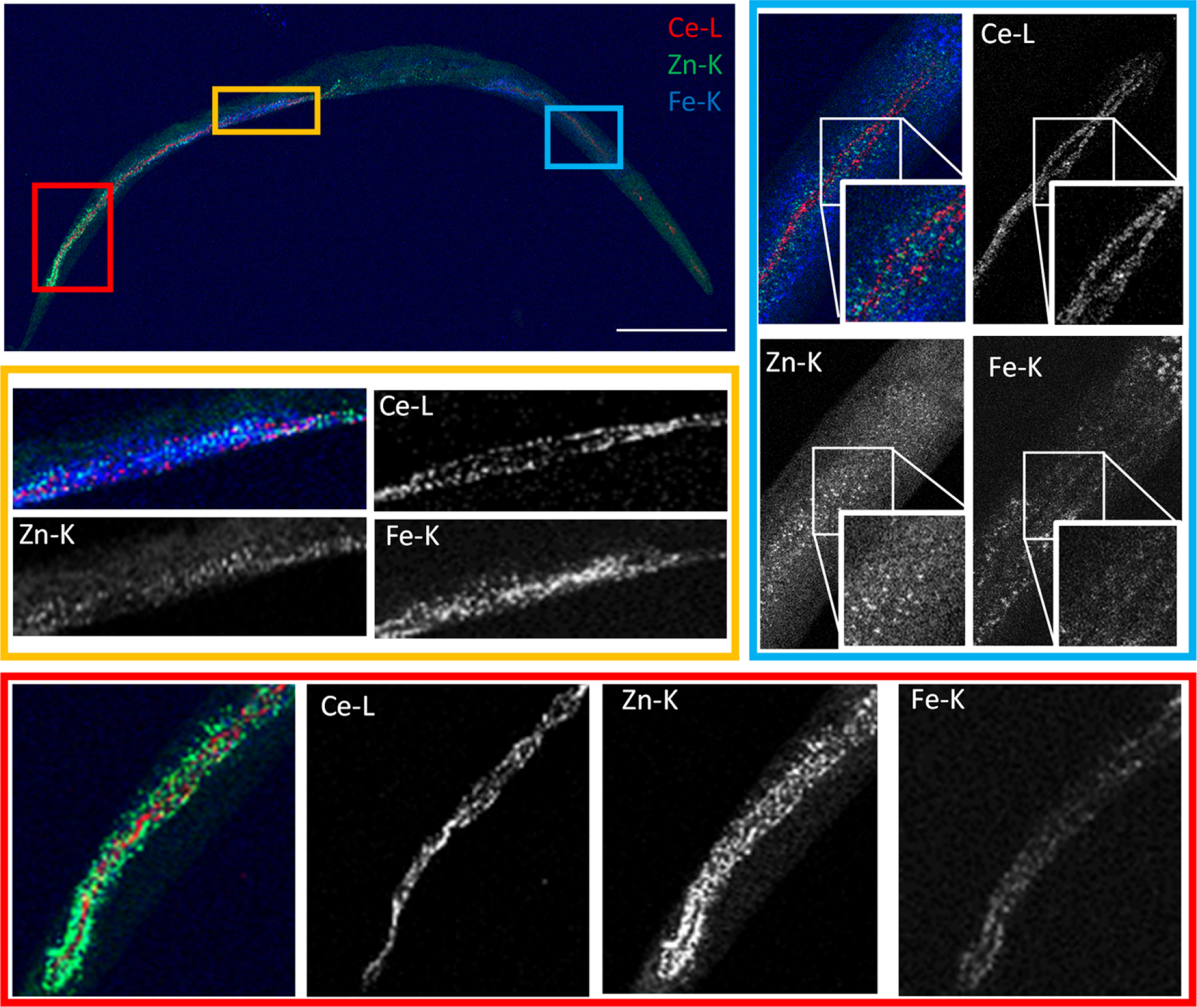
Two-dimensional XRF elemental distribution maps showing Ce accumulations in a depurated (19.5 keV; 1 × 1 μm^2^ step size; 341 × 811 μm^2^ map size; 10 ms exposure/pt) Ce(NO_3_)_3_-exposed nematode, including Ce (red), Zn (blue), and Fe (green) elemental map overlay. Colored boxes on whole nematode maps indicating the regions of interest with detailed Ce, Zn, and Fe elemental maps. Scale bars represent 100 μm.

In line with the ICP-MS measurement of total Ce body burden (Table S2), 2D XRF elemental mapping revealed high retention of Ce from the Ce(NO_3_)_3_ (4.19 mg/) exposure and was primarily associated with the pharynx and the lumen in the depurated nematode (Figure 2). Despite speciation results of the Ce in the exposure media showing a similarly high (92.8 ± 5.2%) particulate fraction as observed by the CeO_2_ NPs (Figure S4), a higher retention of the Ce from Ce(NO_3_)_3_ by the nematodes suggests ionic Ce bound to the negatively charged *E. coli* cell surfaces, with a high degree of LMM Ce dissolution following ingestion.

Despite higher Ce retention by the nematodes from Ce(NO_3_)_3_ than from CeO_2_ NPs, the majority of the Ce was confined within the lumen, thus inferring low levels of intracellular translocation. Competition of Ce with essential elements might influence the degree of uptake and contribute to toxic effects.^43^ Ma et al.^43^ reported chronic toxicity following exposure to Ce^3+^ in the crustacean *Daphnia magna* and hypothesized that this may be the result of competition between the similarly sized Ce^3+^ (ionic radius of 1.01 Å) and Ca^2+^ (ionic radius of 1.00 Å), where Ce^3+^ may replace Ca^2+^ in Ca binding proteins.^44^ Moreover, as Ce^3+^ may interfere with Ca^2+^ transport through the mitochondrial membranes, Ca^2+^ transporters may act as a potential pathway for Ce^3+^ uptake.^44^ However, since we do not see evidence of uptake and translocation of Ce in the current study, results suggest that Ce^3+^ is not easily interchangeable nor interferes with uptake routes for essential elements. Alternatively, it has been suggested that Ce^3+^ may compete with the similarly charged Fe^3+^, where Ce^3+^ may interfere with the oxygen transport by the hemeproteins in *C. elegans*.^45^ Nevertheless, results from the current study do not support this notion, as evident from the lack of Ce uptake into cellular compartments.

The analysis in the current study resulted in a visible Ce signal concentrated in two distinct lines running longitudinally along most of the lumen, with the exception of the left-handed helical twist where only one line is visible in the nematode (Figure 2). This two-line phenomenon is consistent with the elliptical cross section of the intestine and a result of the 3D shape of the lumen, which is associated with higher X-ray attenuation in the 2D projection. The high luminal Ce retention suggests a considerable interaction with the epithelial cell walls and microvilli along the length of the intestine.

### Higher Toxicity from Ce(NO_3_)_3_ Compared to that from CeO_2_ NPs

To further investigate the potential of adverse effects of the Ce exposure to the nematodes, nematodes were analyzed in standard toxicity tests. Exposure to Ce NP induced only minor toxic responses, in agreement with the low retention of the CeO_2_ NPs within nematodes (Figure S5, Tables S1 and S2). However, Arnold et al.^16^ reported that CeO_2_ NPs (53.34 ± 3.12 nm) were more toxic than the equimolar bulk CeO_2_ to *C. elegans*; Arndt et al.^40^ concluded that the level of toxicity toward *C. elegans* was highly dependent on the surface charge of the particles. In the current study, we identified a change from positive (34 mV) to negative (−10 mV) charge of the NPs in ddH_2_O or MHRW, respectively, suggesting a loss of stability in the exposure media (MHRW).^46^ It is thus conceivable that differences in the media composition may have influenced both particle reactivity and interaction with the organism that potentially could affect both retention and toxicity.^40^

The statistically significant (*p* < 0.05, Tukey’s HSD) reduction (60. 6 ± 5.4%) in reproduction observed at 4.37 and 8.03 mg L^–1^ CeO_2_ NP concentrations, but not at the higher concentrations in the current study (Figure S5), supports the hypothesis of substantial agglomeration of the NPs at higher concentrations, further supported by a high degree of visible sedimentation and the speciation results and size distribution measurements (Figures S1, S4, and S6). Similarly, in their CeO_2_ NP toxicity study, Arnold et al.^16^ showed low stability with high aggregation and sedimentation rates in MHRW. As nematodes may, however, ingest particles in the size range of 0.1–3 μm,^47^ it was hypothesized that larger particle aggregates may still contribute to the total exposure, further supported by the observed high uptake of undepurated nematodes in the current study (Table S2, Figure 1). Additionally, reproductive toxicity revealed only low correlation with the LMM Ce fraction in the exposure but a high correlation with the retained Ce fraction (Figure S7), suggesting particle-specific toxicity.

Consistent with the uptake and relatively higher retention measurements, toxicity test results showed statistically significant decreases in growth, fertility, and reproduction for nematodes exposed to Ce(NO_3_)_3_ (Figure S5). This shows that exposure to Ce^3+^ caused adverse effects in a dose-dependent manner, further supporting the notion that the binding of Ce^3+^ to luminal tissues negatively affects physiological functions.

To identify physiological damages from the exposures, microscopy analysis was performed (Figure S8). Histological analysis revealed severe swelling of the intestine posterior to the pharyngeal bulb; however, there were no observable physiological damages to the remaining intestinal structure. Distribution and retention analyses in the current study suggest that the lumen remains functionally intact following either CeO_2_ NP or Ce(NO_3_)_3_ exposure. The fact that we see swelling of the intestine suggested an oxidative stress- or inflammatory-related response, which is consistent with the fact that nanoceria has been associated with redox catalytic ability. We therefore conducted a systematic investigation of the oxidative stress effects of nanoceria compared to ionic Ce in *C. elegans*.

### Impact of Ce on Antioxidant Defenses with No Change in the Cellular Redox Balance in Nematodes

Due to the known pro- and antioxidant properties of Ce, ROS and antioxidant defense production by the nematodes was analyzed. In the current study, an increased *sod-1* gene expression was measured for all CeO_2_ NP concentrations (Figure S9); however, no increase in ROS (Figure S10) or changes in the cellular redox status (Figures S1 and S11) were observed. Contrary to this, results by Zhang et al.^17^ showed high toxicity, ROS accumulation, and oxidative damage by Ce NPs (8 nm) in metal and oxidative stress-sensitive transgenic nematode strains. On the other hand, although a toxic response was measured, Roh et al.^15^ measured no change in antioxidant- and oxidative stress-related genes (including *ctl-2, sod-1*, and *gst-*1) in *C. elegans* exposed to 1 mg L^–1^ CeO_2_ NPs (15–45 nm). It should be noted however that particle size in either study was smaller than that applied in the current study (TEM analysis 10 nm, Figure S1a). Moreover, smaller (15 nm) NPs have been shown to have higher toxicity compared to that of larger (45 nm) Ce particles.^15^

On the other hand, the antioxidant or protective effects of the CeO_2_ NPs in the nematodes were tested by H_2_O_2_-induced ROS production following the exposure toward the NPs (Figure S12). While all nematodes, including controls, showed a statistically significant increase in oxidative stress following the addition of the H_2_O_2_, no difference between controls (no CeO_2_ NPs) or previously CeO_2_ NP-exposed nematodes was found (Tukey’s HSD, *p* > 0.05).

Similar to the CeO_2_ NPs, the exposure to Ce(NO_3_)_3_ led to an increase in the antioxidant defense gene *sod-1*; however, no further increases in the ROS or cellular redox status were observed (Figures S9–S11). Kawagoe et al.^48^ previously reported an oxidative stress response in the mouse liver, in terms of increased metallothionein synthesis and glutathione levels, following the exposure to Ce^3+^, accompanied by significantly decreased growth, fertility, and reproduction. In the current study, increasing concentrations lead to a statistically significant (*p* < 0.05, Tukey’s HSD) reduction in reproduction (Figure S5), coupled with a consistent significantly increased *sod-1* gene expression (Figure S9). However, the lack of further changes in cellular redox balance (Figures S10 and S11) suggests that oxidative stress is not the main toxic MoA of Ce(NO_3_)_3_ in nematodes.

### Ce Retention in the Pharynx Results in Deformities and Increased Antioxidant Defenses

**Figure 3:**
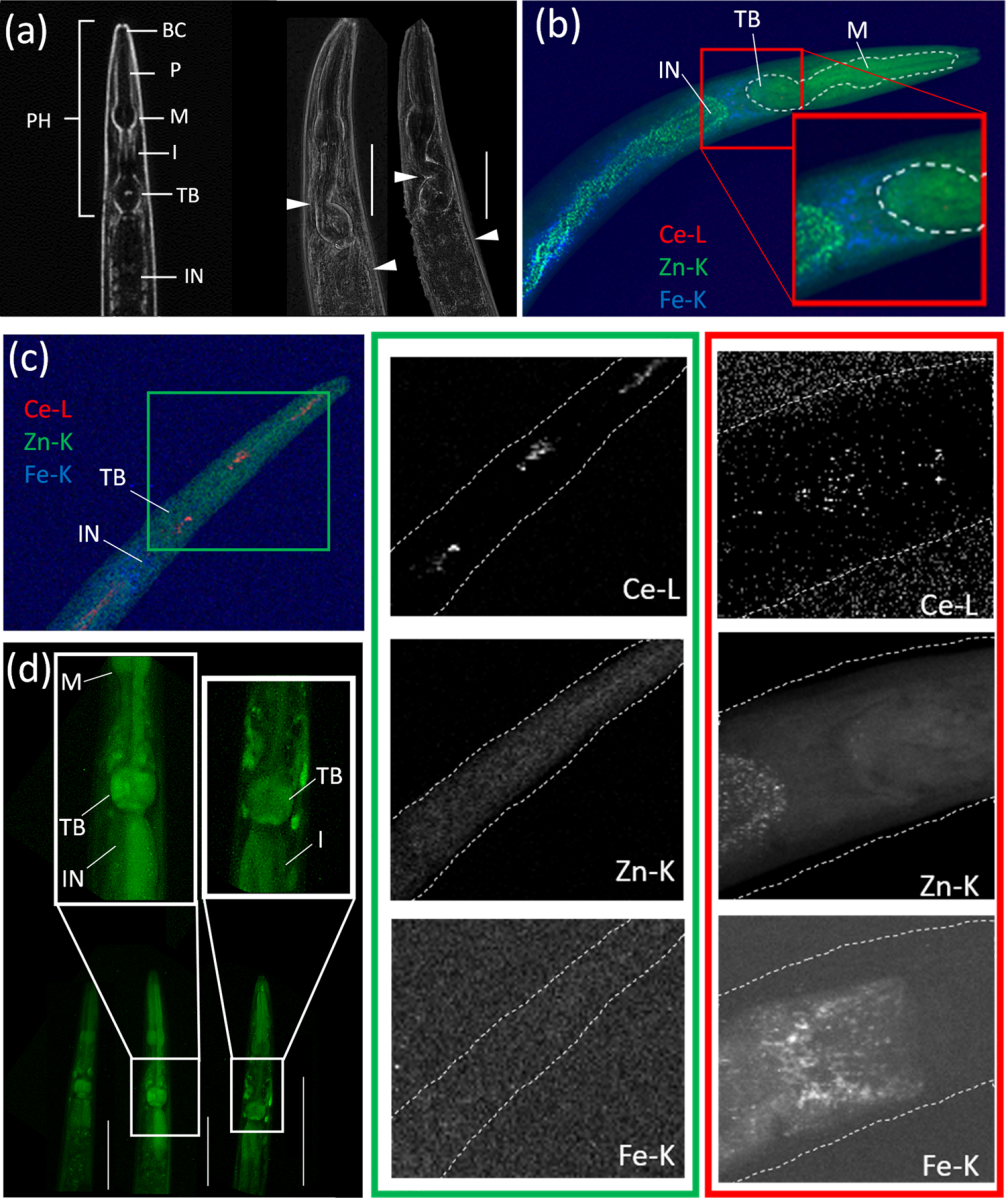
(a) Phase contrast microscope images including annotations of the foregut of a control (left) and CeO_2_ NP (middle)-, or Ce(NO_3_)_3_ (right)-exposed nematode, showing clear deformities of the pharynx in the Ce-exposed nematodes, with visible swelling of the foregut. Buccal cavity (BC), procorpus (P), metacorpus (M), isthmus (I), terminal bulb (TB), pharynx (PH), and intestine (IN). (b) – 2D XRF elemental map of a depurated CeO_2_ NP-exposed nematode (12 keV; 0.5 × 0.5 μm^2^ step size; 561 × 316 μm^2^ map size; 50 ms exposure/pt), including the dotted outline of deformity in the pharynx. The Ce elemental map shows clear retention of Ce around the terminal bulb. (c) 2D XRF elemental map of a depurated Ce(NO_3_)_3_-exposed nematode (19.5 keV; 1 × 1 μm^2^ step size; 341 × 811 μm^2^ map size; 10 ms exposure/pt), with clear retention of Ce in the pharynx around the metacorpus and terminal bulb. Note the low sensitivity of Fe in the nematode due to the recoding at 19.5 keV. (d) Sod-1 expression in the pharynx of a control (Left), CeO_2_ NP (middle), or Ce(NO_3_)_3_ (right) exposed nematode, showing an increased *sod-1* gene expression in the pharynx, particularly associated with the terminal bulb and the foregut. All scale bars represent 100 μm.

Histological image analysis of nematodes following the exposure to either form of Ce revealed a high degree of deformities of the pharynx, particularly in the terminal bulb, and severe swelling posterior to the pharyngeal valve (Figures 3a and S13). Similarly, significant swelling of intestinal cells in *C. elegans* has been observed in a study by Yang et al.^27^ following the exposure to either AgNO_3_ or Cit-Ag NPs and was related to epithelial cell and mitochondria enlargement and shape alterations. Moreover, the exposure to CeO_2_ NPs has been shown to lead to cell death and ROS increases in human lung epithelial cells.^49^ However, Park et al.^49^ proposed that the observed internalization of CeO_2_ NPs into the cells leads to adverse cellular effects. In the current study, however, no significant translocation of the CeO_2_ NPs into the surrounding cells or tissues was observed.

In line with observations of increased deformities within the pharynx, 2D XRF analysis revealed low levels of Ce within and surrounding the terminal bulb following depuration (Figure 3b). The grinder in the terminal bulb has a complex structure designed for grinding food (namely bacteria) before passage into the intestine.^50^ Since pharyngeal activity correlates with food intake, in the abundant presence of food, nematodes may feed at an average rate of 200 pumps per minute.^51,52^ The intensity and high frequency of grinding suggest a considerable mechanical force. Hence, when NP aggregates are ingested alongside bacterial cells, it is plausible that physical damage to the pharyngeal cuticle surrounding the grinder leads to deformation and ROS production. In the current study, the areas of visible foregut deformation, Ce retention, and intestinal swelling correlated to an increase in the *sod-1* gene expression (Figure 3c). Similarly, Zhao et al.^53^ have shown that rapid pumping in early adulthood may lead to mechanical damage to the cuticle of the pharynx, which would lead to the invasion of *E. coli* into the surrounding tissues. Such damages were shown to lead to either death of the nematodes or cuticular healing with associated scarring.^53^ Therefore, it is conceivable that injury, healing, and scarring in this area may result in increased localized ROS formation along with the observed deformation. Moreover, the considerable force applied within the grinder may lead to a subsequent release of smaller particulates or ionic Ce from the NPs and may result in the small, accumulated Ce fraction seen in the 2D-XRF images surrounding the terminal bulb (Figure 3b,c). On the other hand, it is hypothesized that although speciation results from the Ce(NO_3_)_3_ exposure contained a high (81–99.9%) particulate Ce fraction (Figure S4), such agglomerates have low stability and are easily broken up through the grinding motion of the nematodes. Hence, it is conceivable that the Ce(NO_3_)_3_ ingestion resulted in a higher bioavailable fraction, leading to the higher overall measurable retention of Ce in the pharynx (Figure 3c).

To conclude, the current study employed state-of-the-art integrated methods to show that Ce biodistributions coincided with tissue-specific toxic effects, localized tissue deformation, and changes in cellular antioxidant defense. The results presented in the current study demonstrate the utility of integrating SR-based μXRF tissue-specific elemental distribution and adverse effects analysis and represent state-of-the-art methods, providing a robust toxicological evaluation of CeO_2_ NPs.

## Supporting Information Available

Nanoparticle stock preparation and characterization, nematode culture and exposure, CeO_2_ NP characterization, TEM and NTA analysis, sum XRF spectra, speciation data for CeO_2_ NPs and Ce(NO_3_)_3_, XRF maps of unexposed controls, particulate, suspended, and low-molecular mass Ce fractions, uptake data, toxicity test results, DLS size distribution, reproduction vs retained Ce fraction in nematodes, histological analysis, reporter strain *sod-1* (GA508 wuls54[pPD95.77 *sod-1::GFP, rol-6(su1006)*]), HyPer, Grx1-roGFP2 (GRX) and H_2_O_2_, and pharyngal deformities (PDF)
