# Supplementary material for: Synchrotron XRF Analysis Identifies Cerium Accumulation Colocalized with Pharyngeal Deformities in CeO2 NP-Exposed Caenorhabditis elegans
Source: Environ Sci Technol. 2022 Apr 4;56(8):5081–9. doi: 10.1021/acs.est.1c08509 (PMC9022427; doi:10.1021/acs.est.1c08509)
Supplement: Supplementary file 1 — es1c08509_si_001.pdf [file es1c08509_si_001.pdf]

Supporting Information

Synchrotron XRF analysis identifies cerium accumulation co-localized with pharyngeal deformities in CeO_2 NP exposed *Caenorhabditis elegans*

Rossbach Lisa Magdalena[1,2] *, Brede Dag Anders[1,2], Nuyts Gert[3], Cagno Simone. [1,2], Olsson Ragni Maria Skjervold[1,4], Oughton Deborah Helen[1,2], Falkenberg Gerald[5], Janssens Koen[3], Lind Ole Christian[1,2]

[1]Norwegian University of Life Sciences, Faculty of Environmental Sciences and Natural Resource Management, P.O. BOX 5003 NMBU, No-1432 Ås, Norway

[2]Centre for Environmental Radioactivity (CERAD CoE), Faculty of Environmental Sciences and Natural Resource Management, Norwegian University of Life Sciences (NMBU), P.O. Box 5003, 1432 Ås, Norway

[3]University of Antwerp, Faculty of Science, AXIS Research group, Groenenborgerlaan 171, 2020 Antwerp, Belgium

[4]Norwegian University of Science and Technology, Faculty of Natural Sciences, P.O. Box 8900, NO-7491 Trondheim, Torgarden, Norway.

[5]Deutsches Elektronen-Synchrotron DESY, Photon Science, Notkestr. 85, 22607 Hamburg, Germany

*Corresponding Author: Lisa.rossbach@nmbu.no

Number of pages: 19

Number of Figures: 13

Number of Tables: 2

1. Method

1.1 Nanoparticle stock preparation and characterization

The CeO_2 NP (544841, Sigma Aldrich) stock suspensions were prepared by adding 10 mg L^{-1} CeO_2 NPs to 10 ml ddH_2O (15 MΩ·cm) with 0.2 % Tween 20 (P1379, Sigma Aldrich). The suspensions were vortexed at 3000 rotations/min (MS basic, IKA) for 5 minutes, before being alternately sonicated at 35 kHz (Sonorex RK106, Bandelin) for 5 minutes and vortexed for 10 seconds, for 1 hour. Three replicate working solutions were prepared by diluting 1 ml of the NP suspension 10 x in moderately hard reconstituted water (MHRW, pH 7.7) [1] with 0.2 % Tween 20 (MHRW-T). The aforementioned alternation of vertexing and sonication was repeated for the preparation of the MHRW-T. The working solutions were allowed to settle over night to remove larger aggregates from the suspensions. Aliquot samples were taken from the 5 ml mark, and immediately used for toxicity testing or characterization. For the purpose of comparison, the $Ce(NO_3)_3$ stock solution was prepared in MHRW + Tween 20.

Size distribution of the NPs was measured using Transmission electron microscopy (TEM, Morgagni 268, FEI), Nanoparticle tracing analysis (NTA, Nanosight LM10, Malvern Panalytical), and dynamic light scattering (DLS, Malvern PN3702 Zetasizer Nanoseries, Malvern Inc., Malvern, UK). Image analysis software ImageJ was used for maximum ferret diameter measurements of TEM micrographs. For electrophoretic mobility measurements of the NPs, particles suspensions were measured using dynamic light scattering (DLS, Malvern PN3702 Zetasizer Nanoseries). Size fraction and particle dissolution assessment was carried out at T-0 and 72 hrs of exposure by ultrafiltration (14,000 *g* for 30 min) of the exposure media using pre-conditioned <3 kDa Millipore Centrifugal filters (Amicon, Millipore, Billerica, MA) of low, medium, and a high $Ce(NO_3)_3$ or CeO_2 NP concentration, in triplicate. The contents of the whole exposure well was taken, to include all particles. Prior ultrafiltration samples were centrifuged (2000 g for 5 minutes) to remove the *Escherichia coli* and larger aggregates to avoid clogging of the filter units. Exposure concentrations and size fractions were determined using Inductively coupled mass spectroscopy (ICP-MS, ICP-MS Agilent 8800, Mississauga, Canada) measuring the Ce (140) isotope, at a detection limit of 0.0003 ppm. A house standard (1643H), prepared from the certified refence material 1640a with the addition of a 1 µg L^{-1} Ce from the CRM 71A, was used as a calibration check. A purity check of the CeO_2 NP stock solution showed 97.1% purity of the CeO_2 NPs in dd$H_2$0.

1.2 Nematode Culture and Exposure

For synchronization of the nematode culture, nematodes previously kept in liquid cultures were treated with hypochlorite solution for egg extraction. Eggs were allowed to hatch over night to obtain synchronized L1 stage nematodes. Synchronized nematodes were exposed for 96 h, in triplicate, in 24 well exposure plates, from L1 stage at 20°C in the dark, with continuous gentle shaking (100 rotations/min). Each well contained 1 ml of *Escherichia coli* OP50 resuspended in MHRW [1], and 11 ± 3 L1 stage nematodes. The N2 Bristol strain was exposed to seven concentrations in the range of 0.5 – 34.96 mg Ce L^{-1}, or 12 concentrations in the range of 2.3 – 26 mg Ce L^{-1} of CeO_2 NPs or $Ce(NO_3)_3$, respectively (measured concentrations, see table S1). For the termination of the N2 toxicity test at T-96 h, 500µl of Rose Bengal (616 µM) was added to each well, before heating at 80°C for 10 minutes. For the assessment of growth, fertility and reproduction [2], a stereo microscope (Leica M205C), equipped with digital camera (MC170 HD, Leica), and a hand held tally counter were used.

To determine the uptake of Ce, nematodes were exposed in triplicate to either 2.3, 4.19, and 614, or 0.99, 8.03, and 34.96 mg Ce L^{-1} of either $Ce(NO_3)_3$ or CeO_2 NPs, respectively, for 72 hours. For total body burden nematodes were removed from the exposure and washed three times in 1 ml MHRW (un-depurated nematodes). Parallel replicate nematodes were placed on NGM agar plates seeded with *E. coli*, to assess the Ce fraction tightly bound to the nematodes. Following 2 hours of depuration, nematodes were hand picked off the NGM plates and transferred into 15 ml tubes (depurated nematodes). Supernatant from all samples was evaporated prior ultrapure HNO_3 + 0.02% HF acid digestion at 90°C for 2 hours. To identify possible transfer of Ce from the supernatant during transfer, blank samples containing no nematodes were taken in addition. Samples were diluted to 10 vol % HNO_3 prior ICP-MS analysis. For both, un-depurated and depurated nematode samples, exact numbers of nematodes per individual tubes were recorded, for total Ce/nematode calculations.

For the *in vivo* assessment of the Sod-1, HyPer, and GRX strains, nematodes were sampled at 72 h of exposure. Samples were analyzed for fluorescence as previously described by Rossbach et al. [3] and Maremonti et al. [4]. Briefly, nematodes were immobilized using 30 mM sodium azide (NaN_3) and imaged on a fluorescent microscope (Leica DM6 B) equipped with a 405 nm excitation and 535 emission filter. For the oxidized to reduced ration of the HyPer and GRX biosensor strains, a second image at excitation 490 nm and emission 535 nm was taken. Ratio calculations were conducted as described by Back et al. [5]. Approximately 10 nematodes were

images per triplicate exposure well. Additionally, nematodes were imaged in phase contrast mode at 10x magnification to assess the total body length of the nematodes. For Quantification of fluorescent measurements, the Leica Application Suit X image software was used, for pixel based average intensity. To account for impacts of developmental stage on the SOD-1 signal strength, SOD-1 average intensity was normalized to total body length [6].

To test the antioxidant properties of the CeO_2 NPs, the aforementioned Ce exposed GRX nematodes were subjected to a further 10 min acute H_2O_2 (23613.366 VWR Chemicals) exposure (107.7, 213.4, or 323.1 mM). Sampling was conducted as previously described.

2. Results

CeO_2 NP characterization

Several particle characteristics, including electrophoretic mobility and aggregation, are sensitive to small changes in pH, conductivity, or media composition, and may affect the stability of the particles in the exposure [7]. Therefore, to explain uptake, retention, and toxic effects of NPs, detailed characterization of the particles in their pristine state and in the exposure media is of importance [8].

To monitor the behavior of cerium during the exposure, concentrations in the exposure media were quantified by ICP-MS at T - 0 and 72 h (Table S1). Size distributions were relatively constant at 0 and 72 hr with the majority of the Ce being found in the particulate fraction, assumed to be associated with the *E. coli* bacterial cells. The sharp increase in the suspended fraction at 34.96 mg/l is most likely due to a sampling error. Results confirmed no significant difference between measured and nominal concentrations for the $Ce(NO_3)_3$ exposure at the start (T-0) of the exposure (Table S1). However, a 35 – 80 % reduction in total Ce concentration was measured at 72 h in the $Ce(NO_3)_3$ exposure. In comparison, CeO_2 NP concentrations remained relatively stable with a 0.4 – 22 % reduction over the course of the 72 hours exposure period (Table S1). The high degree of Ce removal from the $Ce(NO_3)_3$ exposure is hypothesized to be the results of adhesion of the Ce to the exposure well surface.

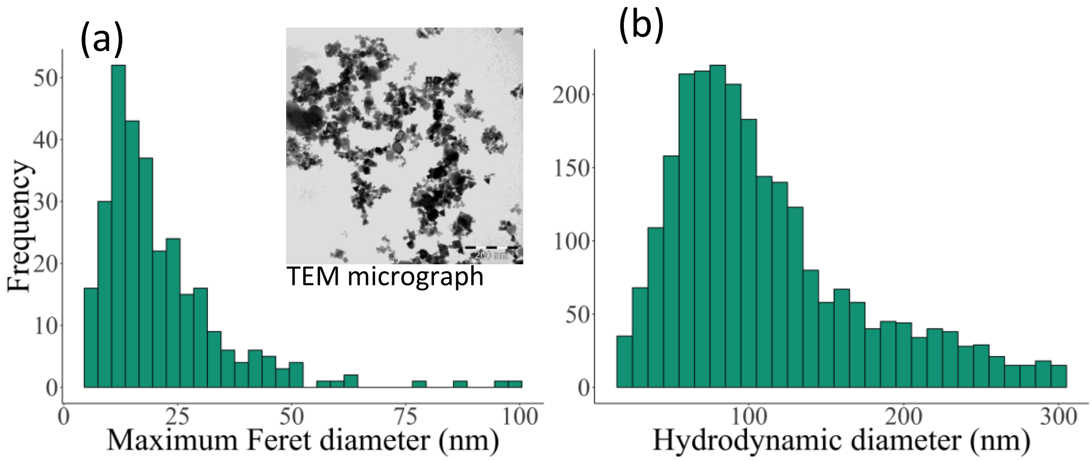

Figure S1: Histograms of the size distribution of CeO_2 NPs measured using TEM (a) and NTA (b). Note the different range of x-values for the two graphs. The maximum Ferret diameters in (a) are measured from N=300 NPs from 5 TEM micrographs. The hydrodynamic diameters in (b) are measured from 5 videos of 60 seconds, and an upper cut-off at 300 nm was set when plotting the results.

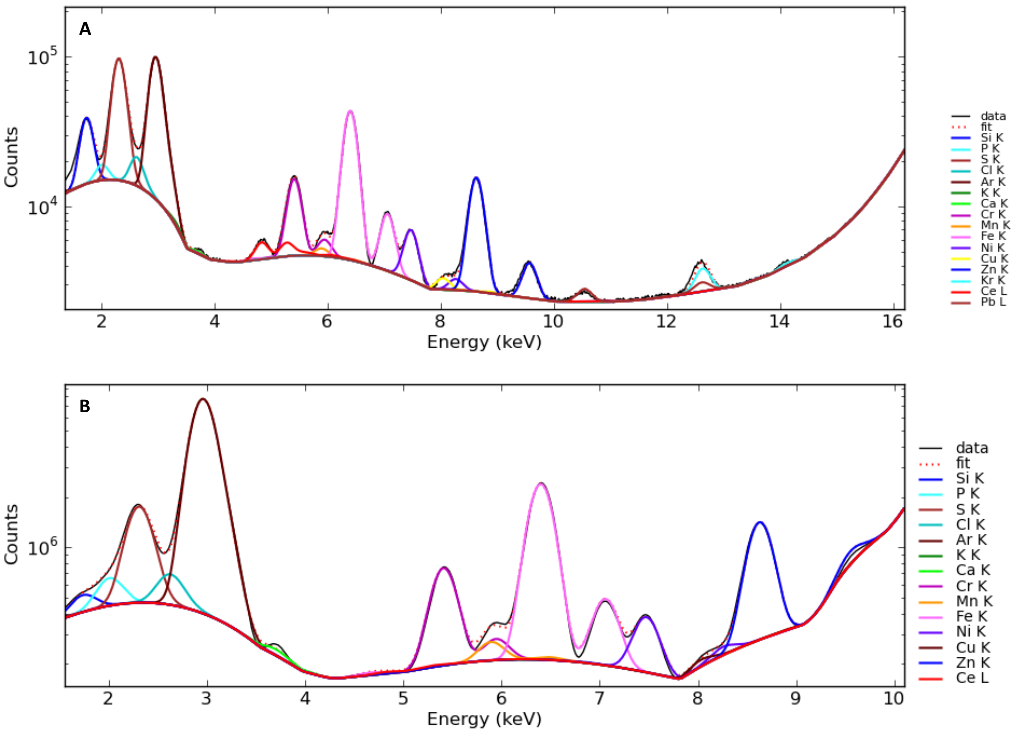

Figure S2: Sum XRF spectra of maps of whole intact *Caenorhabditis elegans* exposed to either $Ce(NO_3)_3$ (A) or CeO_2 NPs (B) obtained at the microprobe end-station of the P06 Hard X-ray Micro/Nano-Probe beamline of the PETRA III storage ring of the DESY facility (Hamburg, Germany).

Table S1: Speciation data for $Ce(NO_3)_3$ or CeO_2 NPs in MHRW + Tween at the start (T – 0) and the end (72 hours) of exposure. Total, suspended, and <3 kDa fraction were analyzed using ICP-MS, while the particulate fraction was calculated as the difference between the total and suspended fraction.

		T-0							72 hours						
		**Total**		**Suspended**		**<3k Da**		**Particulate**	**Total**		**Suspended**		**<3k Da**		**Particulate**
**Exposure**	**Nominal concentration (mg/L)**	conc (mg/L)	SD	conc (mg/L)	SD	conc (mg/L)	SD	Calculated (mg/l	conc (mg/L)	SD	conc (mg/L)	SD	conc (mg/L)	SD	Calculated (mg/l)
**Control**		0,00019	0,00004	0,00000	0,00000	0,00000	0,00001	0,00019	0,00025	0,00009	0,00022	0,00021	0,00014	0,00015	0,00003
**$Ce(NO_3)_3$**	**2**	2,29809	0,30279	0,03584	0,00459	0,00008	0,00004	2,26225	1,48625	1,05547	0,00569	0,00201	0,00022	0,00020	1,48056
	**4**	4,18789	0,12378	0,05911	0,05964	0,00002	0,00001	4,12878	N/A	N/A	0,02037	0,01334	0,00058	0,00078	N/A
	**6**	6,14161	0,28008	0,58100	0,52821	0,00006	0,00002	5,56061	2,08925	0,85947	0,23719	0,29517	0,00008	0,00008	1,85206
**CeO_2 NPs**	**1.03**	0,99128	0,01215	0,12702	0,01455	0,00003	0,00005	0,86425	1,0144	0,01288	0,01107	0,00076	0,00027	0,00024	0,09037
	**8.25**	8,02740	0,12549	0,64659	0,08292	0,00007	0,00003	7,38082	6,3242	0,07758	0,11131	0,02129	0,00003	0,00003	0,52110
	**33**	34,95596	0,42262	1,57788	0,11983	0,00005	0,00006	33,37808	34,83600	1,96401	20,54884	0,74131	0,35360	0,49998	14,28715

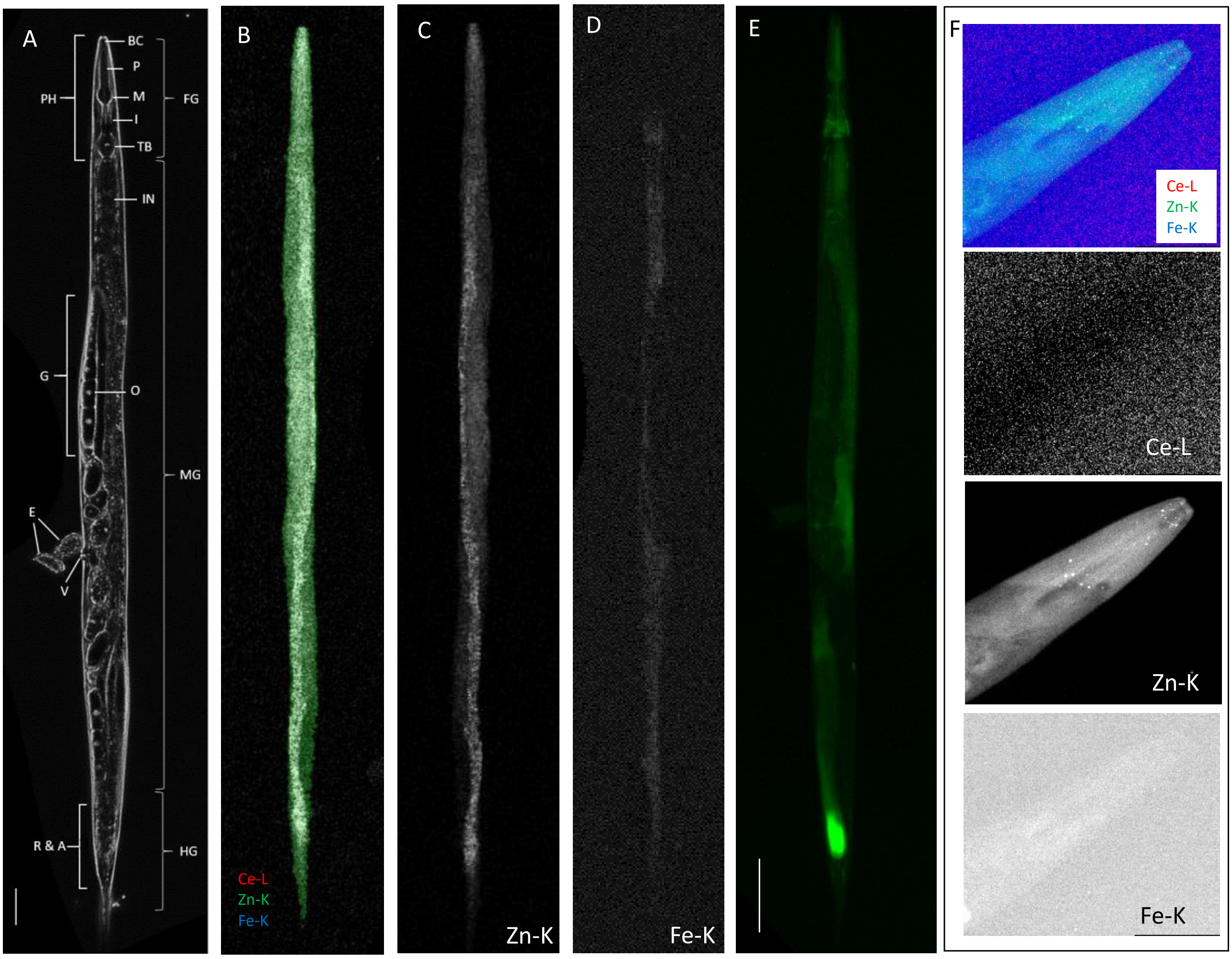

Figure S3: Imaging of an unexposed nematode (control). (A) Phase contrast (PH) microscopy image showing nematode anatomy. The foregut (FG), midgut (MG), hindgut (HG), buccal cavity (BC), procorpus (P), metacorpus (M), isthmus (I), terminal bulb (TB), pharynx (PH), intestine (IN), gonads (G), oocytes (O), vulva (V), rectum (R), anus (A), and eggs (E). (B - D) 2D XRF map (19,5 keV, 1 x 1 μm^2, step size, 7 msec exposure/pt), elemental maps; (B) Ce, Zn, and Fe (composition map), (C) Zn and (D) Fe. (E) *sod-1* gene expression of an unexposed control nematode. (F) 2-D XRF elemental maps of the nematode pharynx showing the nerve ring. Scale bars represent 100 μm.

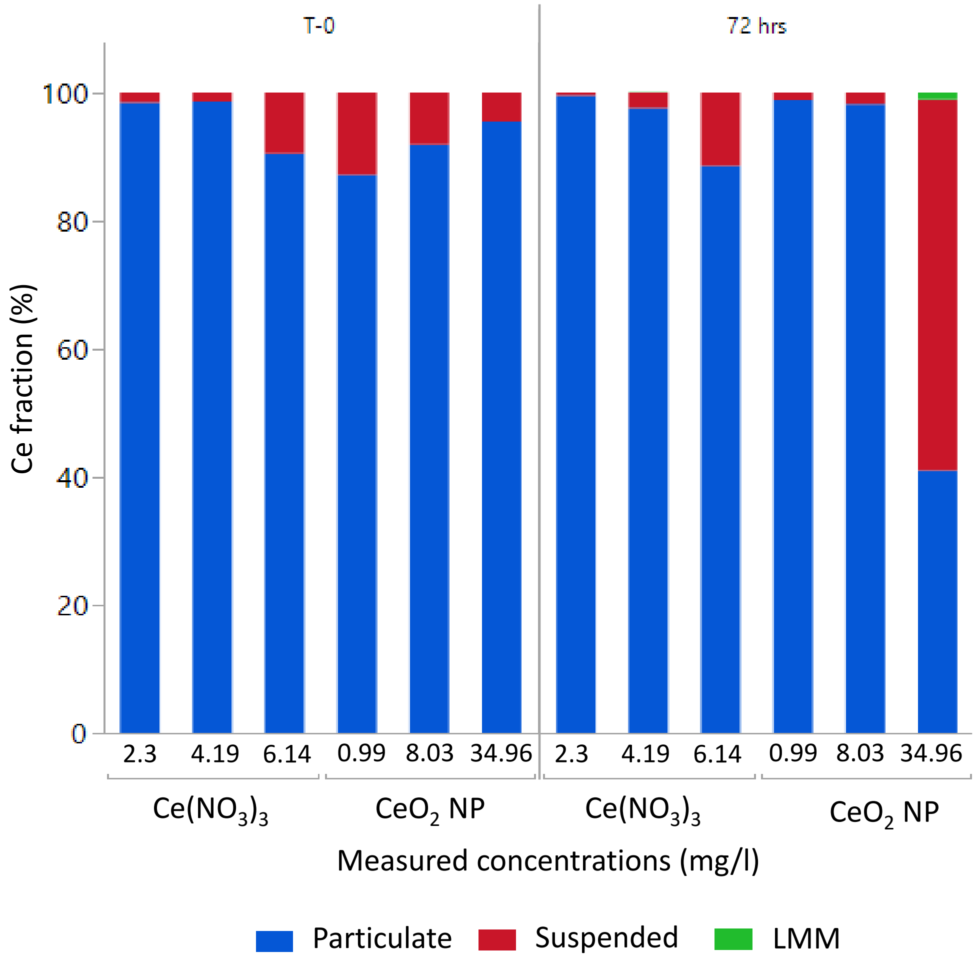

Figure S4: Particulate, suspended, and low molecular mass (LMM, <3 kDa) Ce fraction in the exposure media containing either $Ce(NO_3)_3$ or CeO_2 NPs at T-0 or 72 hours in MHRW containing *E. coli*. For the suspended fraction *E. coli* was removed via centrifugation.

Table S2: Measured Ce concentrations in undepurated and depurated nematodes (n = 21 ± 9) for either $Ce(NO_3)_3$ or CeO_2 NPs exposed for 72 hours in MHRW*.

	Ce concentrations exposure media		Ce concentrations in nematodes	
Exposure	Nominal (mg/l)	Measured (mg/l)	Undepurated (µg/nematode)	Depurated (µg/nematode)
$Ce(NO_3)_3$	2	2.3 ± 0.3	0.1 ± 0.05	0.01 ± 0.004
	4	4.19 ± 0.12	0.09 ± 0.04	0.06 ± 0.004
	6	6.14 ± 0.28	0.09 ± 0.02	0.04 ± 0.01
CeO_2 NPs	1.03	0.99 ± 0.12	0.003 ± 0.0007	<LOD
	8.25	8.03 ± 0.13	0.04±0.01	0.02 ± 0.02
	33	34.96 ± 0.42	0.04 ± 0.02	0.004 ± 0.0008

*LOD = limit of detection = 0.0003 ppm.

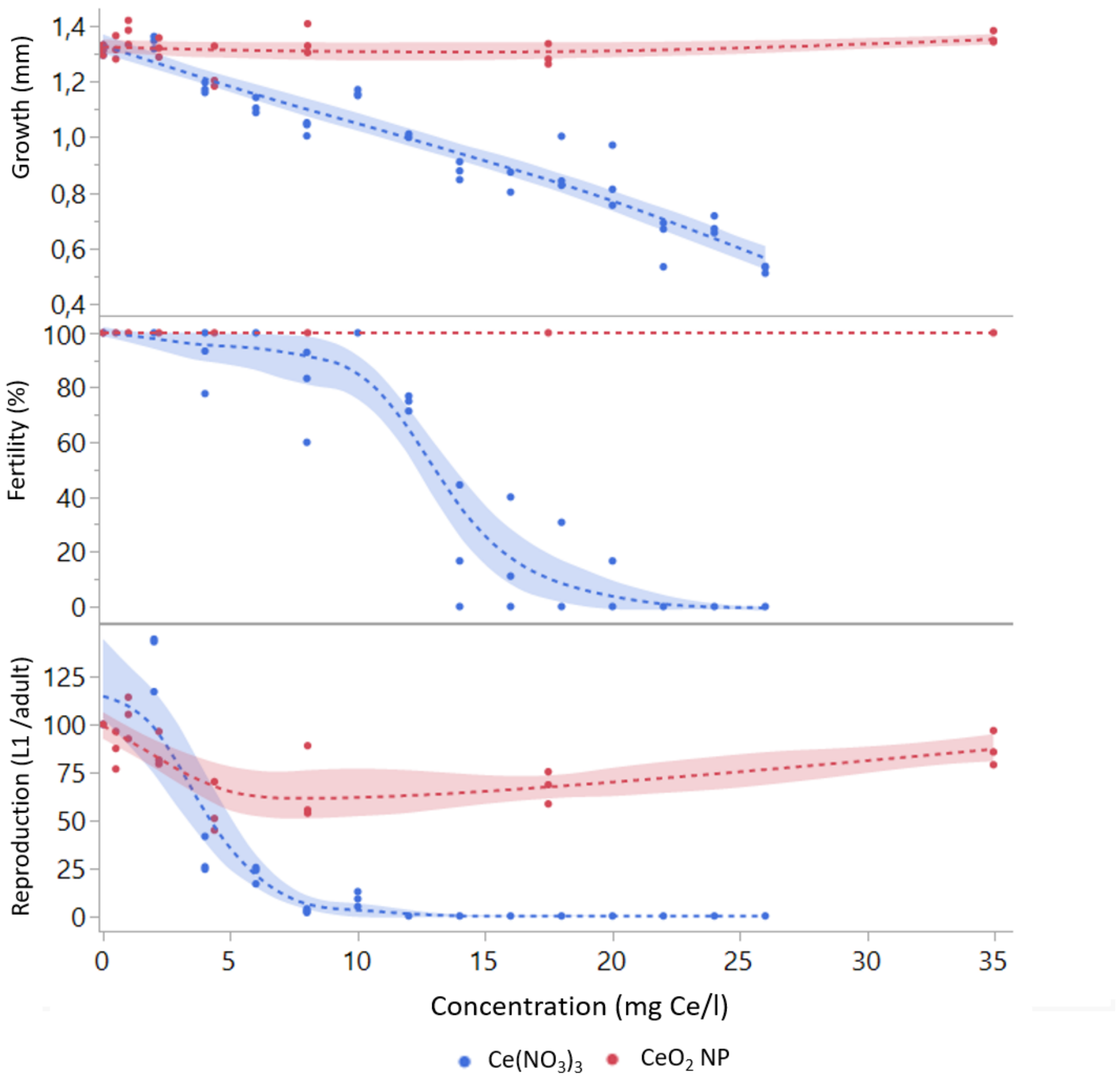

Figure S5: Growth, fertility and reproduction of *C. elegans* following the exposure to either $Ce(NO_3)_3$ (a) or CeO_2 NPs (b) (mean ± SD) in MHRW containing *E. coli* in a standard 96 hr toxicity test.

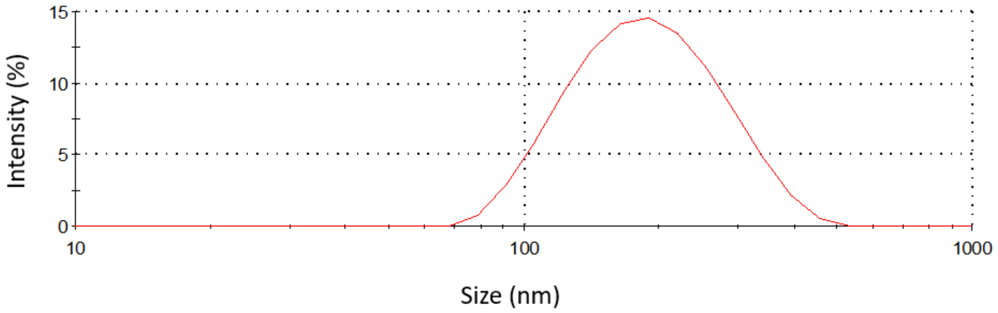

Figure S6: Size distribution of the CeO_2 NPs in the stock suspension (MHRW-T), as measured by dynamic light scattering (5 measurements, 10 runs each).

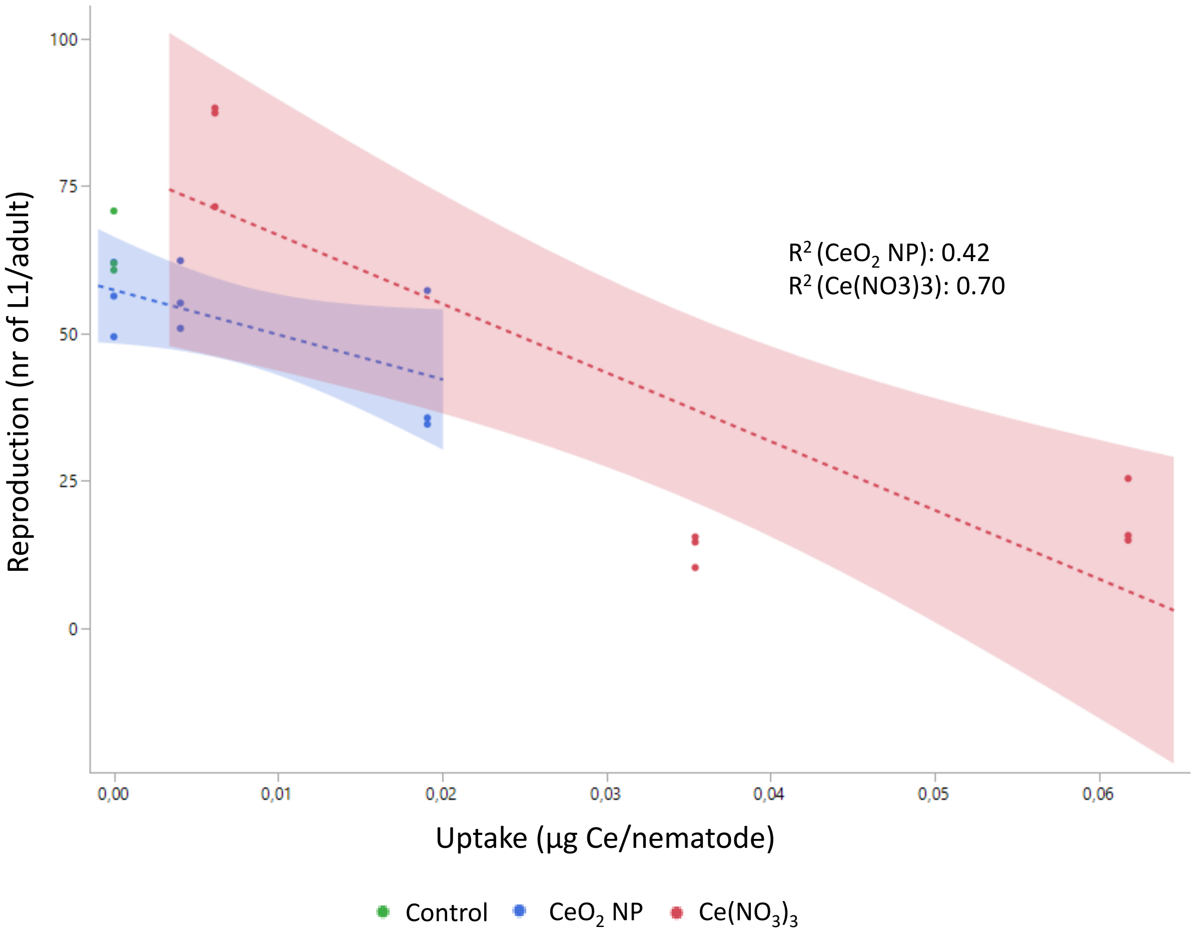

Figure S7: Measured reproduction (nr of L1s/hermaphrodites) as a function of Ce uptake (retained Ce/nematode follow depuration) with lines of best fit.

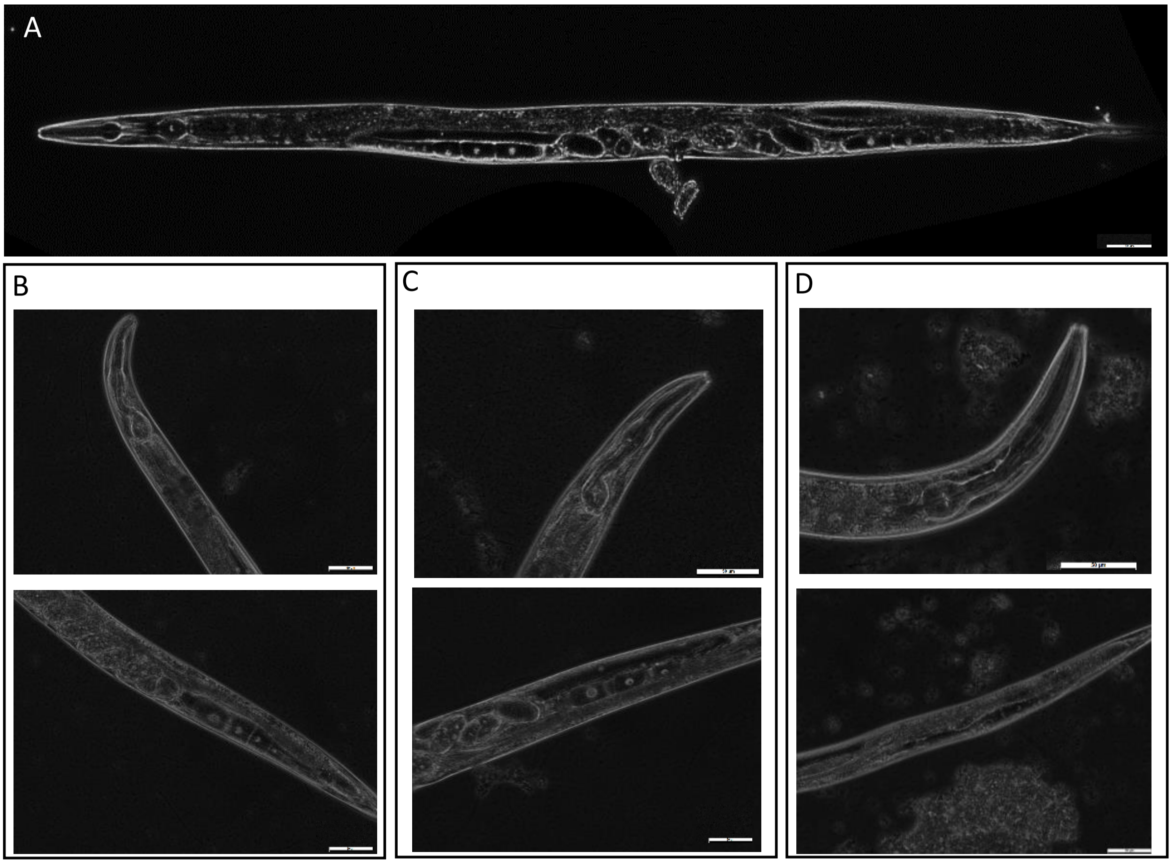

Figure S8: (A) Histological analysis of whole body control, or pharynx and posterior gonads, eggs, and intestine of (B) 17.48 mg/l or (C) 8.03 mg/l CeO_2 NP, or (D) 6.14 mg/l $Ce(NO_3)_3$ exposed *C. elegans.* All scale bars represent 50 µm.

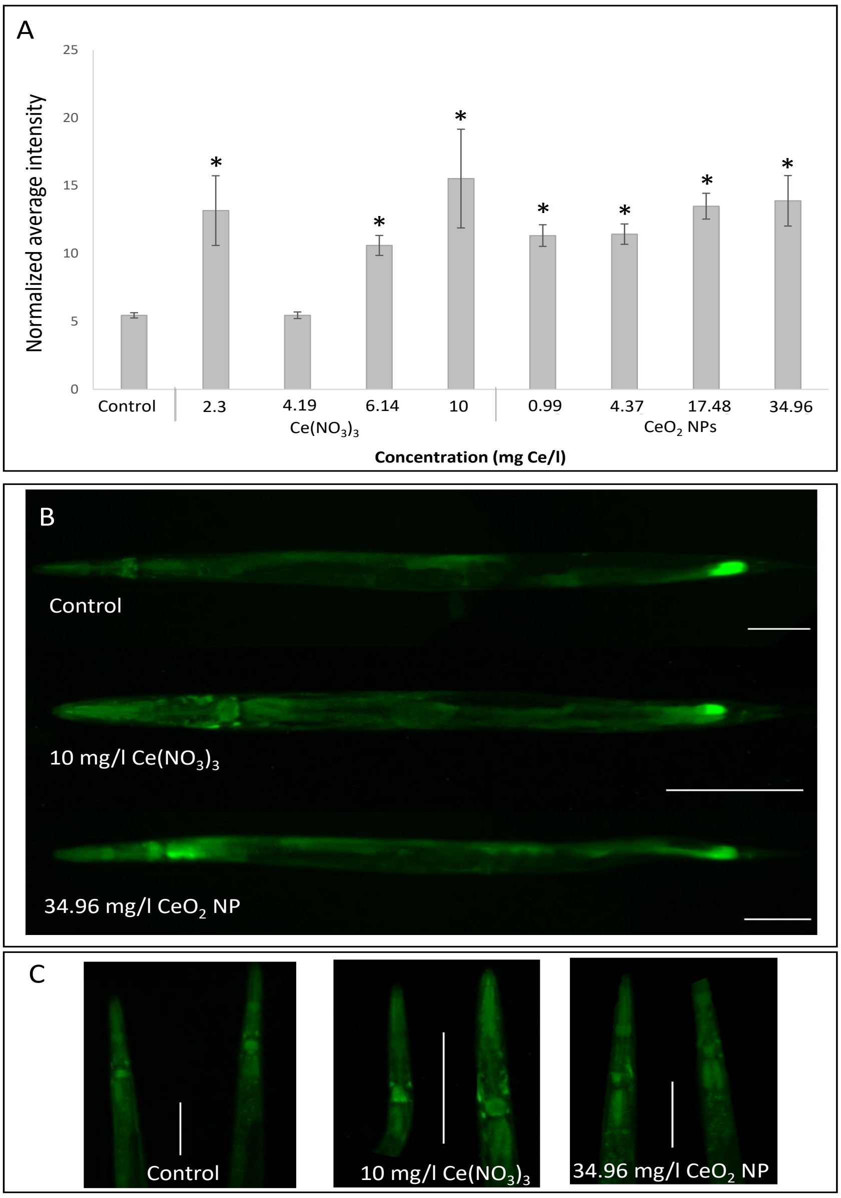

Figure S9: (A) Normalized average intensity measurements from the Sod-1 nematode strain after exposure to either $Ce(NO_3)_3$ or CeO_2 NPs, and (B) representative Sod-1 nematode images, with (C) close up images of the pharynx for two individuals from 3 different treatments. Scale bars represent 100 µm. Asterisk (*) indicate a significant difference ($p < 0.05$ students t-test) between the exposure group and control.

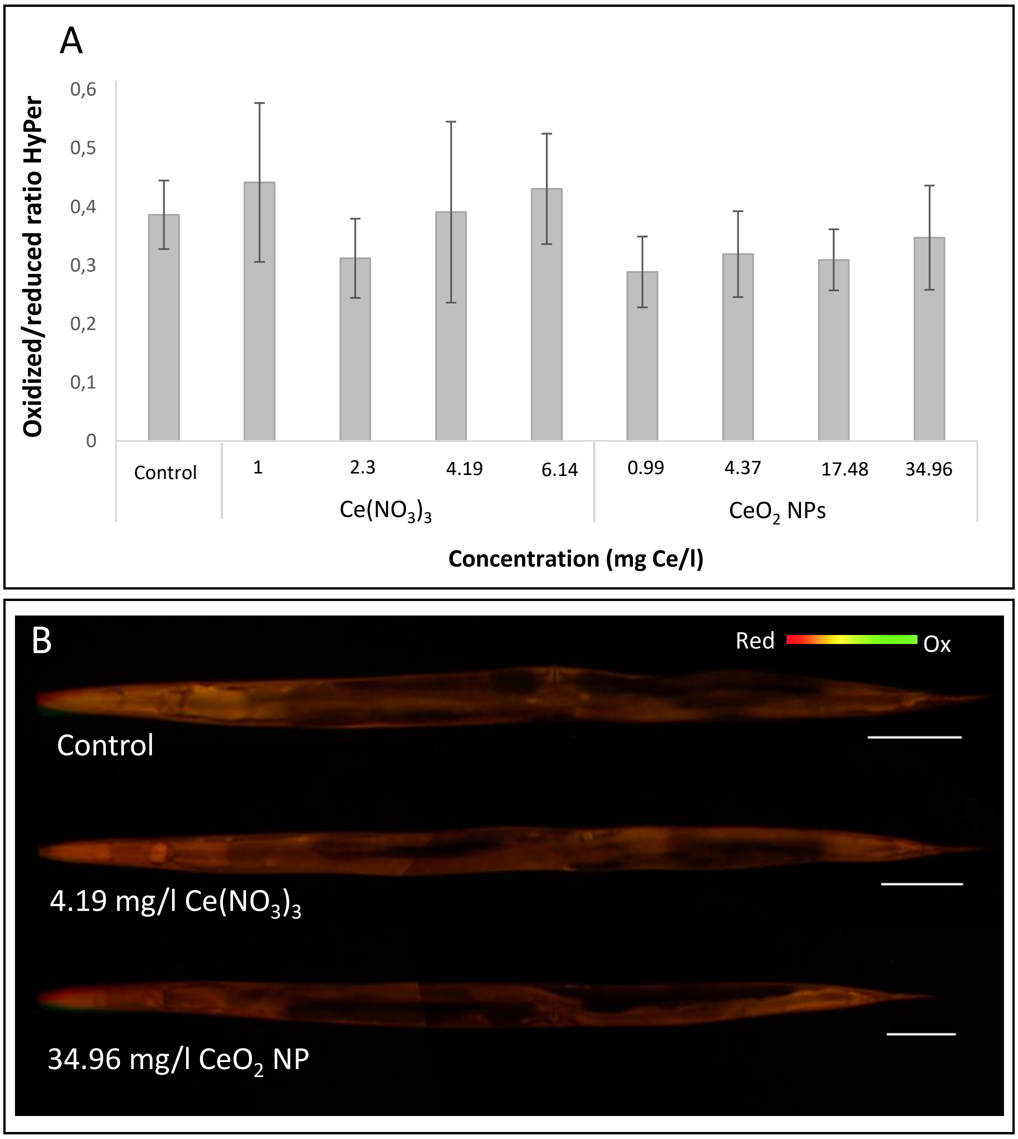

Figure S10. (A) Oxidized to reduced ratios of the hydrogen peroxide biosensor HyPer measured i*n vivo* in response to the exposure to either $Ce(NO_3)_3$ or CeO_2 NPs (n = 10 per concentration), with (B) corresponding representative images (n = 10 per concentration). Scale bars represent 100 µm.

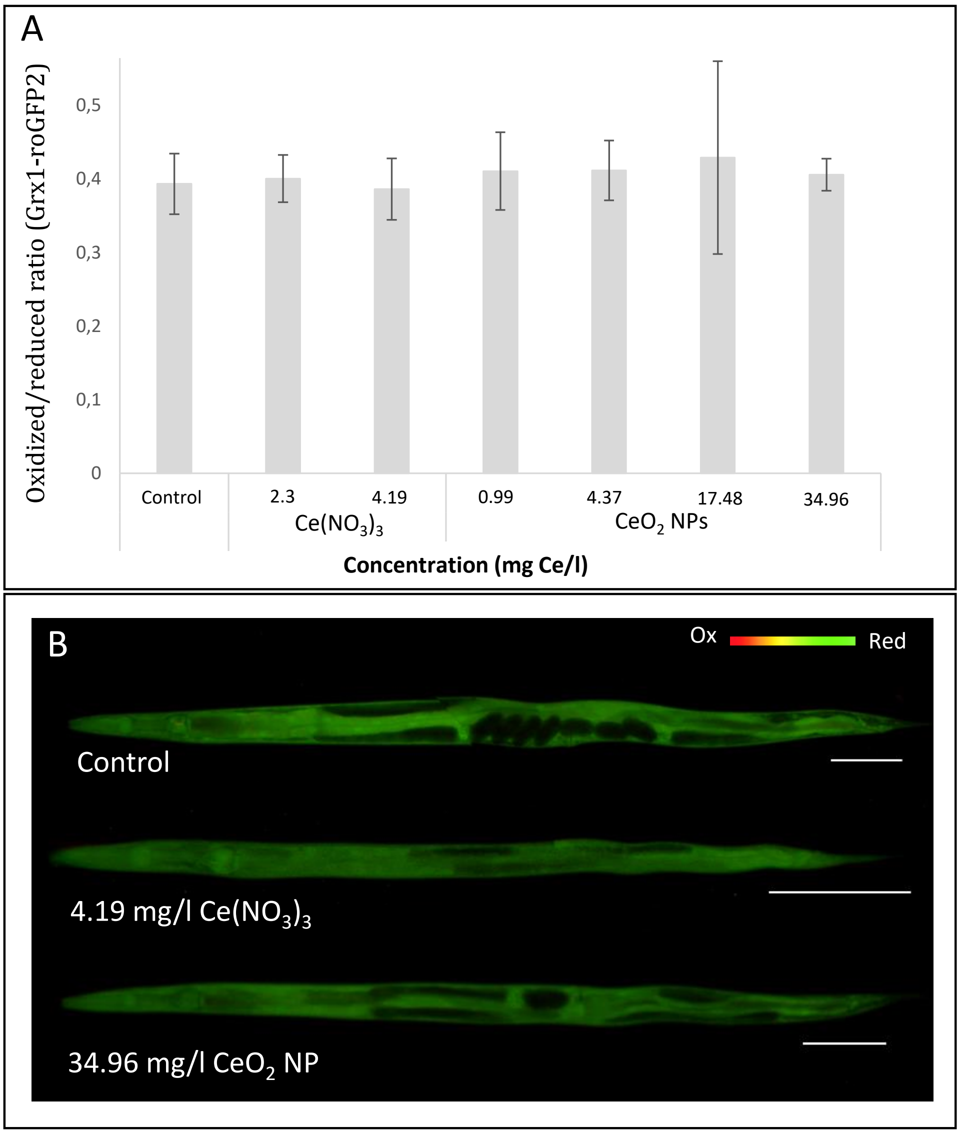

Figure S11: Oxidized to reduced ratios of the biosensor Grx1-roGFP2 (A) with corresponding representative images (B), measured *in vivo*, in response to the exposure to either $Ce(NO_3)_3$ or CeO_2 NPs (n = 10 per concentration). Scale bars represent 100 µm.

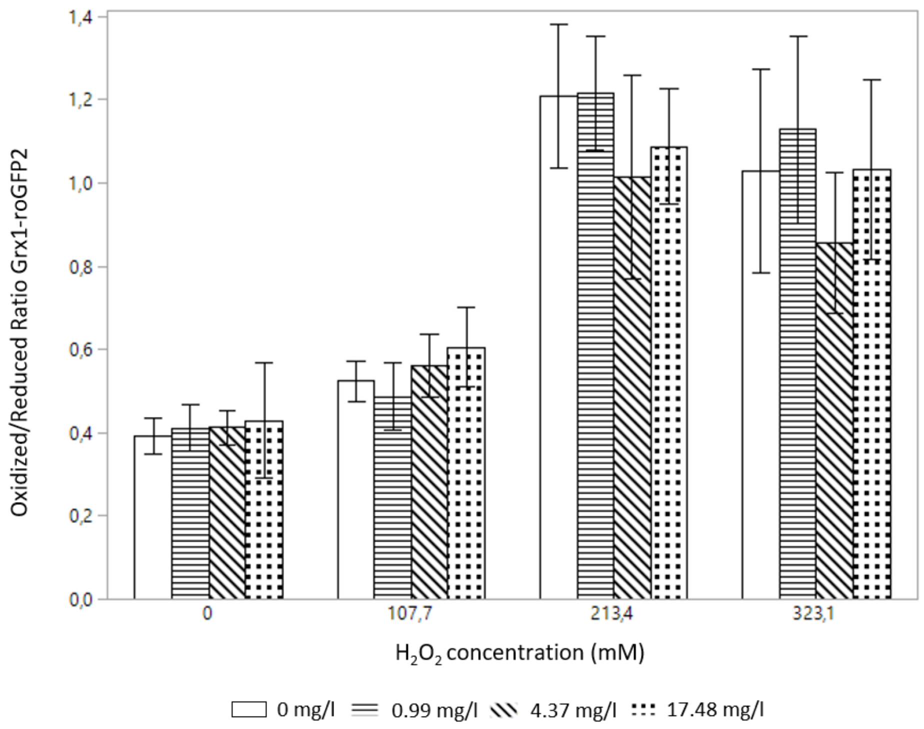

Figure S12: Oxidized to reduced ratios of the biosensor Grx1-roGFP2 measured *in vivo,* in response to a chronic 72 hour CeO_2 NP (0, 0.99, 4.37, or 17.48 mg Ce/l) exposure with a subsequent acute (6 min) exposure to H_2O_2 (0, 107.7, 213.4, or 323.1 mM) (n = 10 nematodes per concentration).

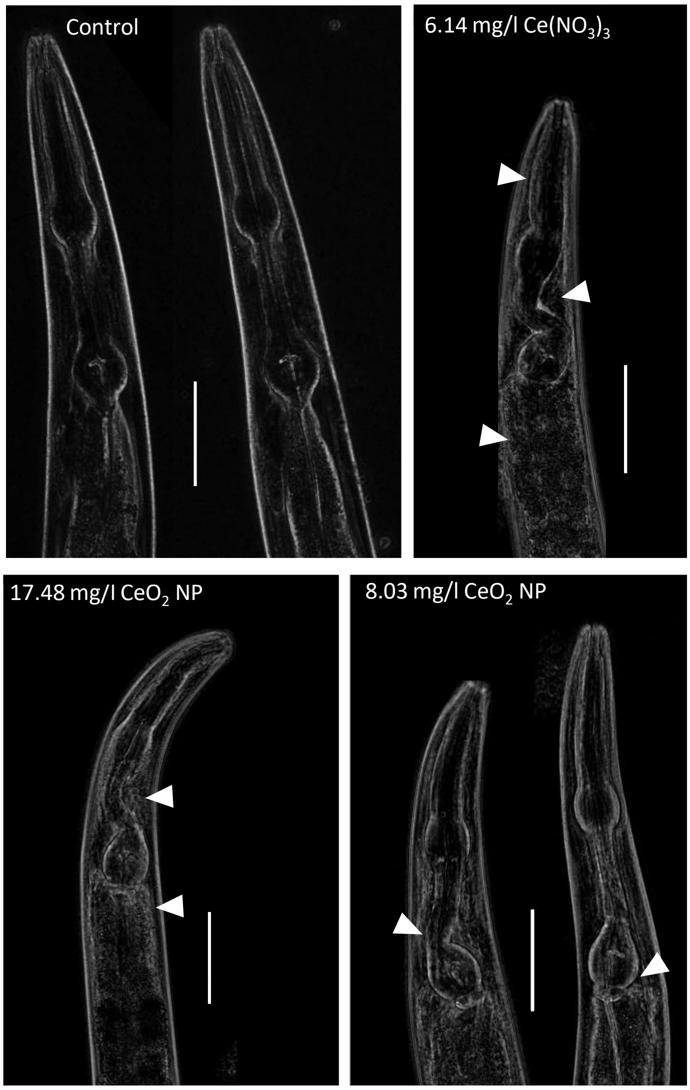

Figure S13: Pharyngal deformities of N2 strain *C. elegans* exposed to either $Ce(NO_3)_3$ or CeO_2 NPs for 72 hours in MHRW. Arrows indicate deformities observed in nematodes exposed to either $Ce(NO_3)_3$ or CeO_2 NPs. Scale bars represent 100 µm.

References:

1. United States Environmental Protection Agency, U.E., *Methods for Measuring the Acute Toxicity of Effluents and Receiving Waters to Freshwater and Marine Organisms, 5th edition. EPA-821-R-02-012. Office of water, Washington DC, USA.* 2002.
2. ISO, *Water quality -- Determination of the toxic effect of sediment and soil samples on growth, fertility and reproduction of Caenorhabditis elegans (Nematoda).* ISO 10872:2010. Geneva, Switzerland., 2010.
3. Rossbach, L.M., Oughton, D. H., Maremonti, E., Coutris, C., and Brede, D. A. *In vivo assessment of silver nanoparticle induced reactive oxygen species reveals tissue specific effects on cellular redox status in the nematode Caenorhabditis elegans.* Sci. Total Environ., 2020. **721**: p. 137665.
4. Maremonti, E., Eide, D. M., Rossbach, L. M., Lind, O. C., Salbu, B., and Brede, D. A. *In vivo assessment of reactive oxygen species production and oxidative stress effects induced by chronic exposure to gamma radiation in Caenorhabditis elegans.* Radic Bio., 2019.
5. Back, P., De Vos, W. H., Depuydt, G. G., Matthijssens, F., VanFleteren, J. R., and Braeckman, B. P. *Exploring Real-Time in vivo Redox Biology of Developing and Aging Caenorhabditis elegans.* Radic. Bio., 2012. **52**(5): p. 850-859.
6. Doonan, R., McElewee, J. J., Matthijssens, F., Walker, G. A., Houthoofd, K., Back, P., Matscheski, A., VanFleteren, J. R., and Gems, D. *Against the Oxidative Damage Theory of Aging: Superoxide Dismutases Protect Against Oxidative Stress but have Little or no Effect on Life Span in Caenorhabditis elegans.* Genes Dev., 2008. **22**(23): p. 3236-3241.
7. Kosmulski, M., *The pH dependent surface charging and points of zero charge. VII. Update.* Adv. Colloid Interface Sci., 2018. **251**: p. 115-138.
8. Kleiven, M., Rossbach, L. M., Gallego-Urrea, J. A., Brede, D. A., Oughton, D. H., and Coutris, C. *Characterizing the behavior, uptake, and toxicity of NM300K silver nanoparticles in Caenorhabditis elegans.* Environ. Toxicol. Chem., 2018. **37**(7): p. 1799 - 1810.
